# Evaluating an educational intervention to improve the accuracy of death certification among trainees from various specialties

**DOI:** 10.1186/1472-6963-7-183

**Published:** 2007-11-15

**Authors:** Jesús Villar, Lina Pérez-Méndez

**Affiliations:** 1 Multidisciplinary Organ Dysfunction Evaluation Research Network (MODERN), Research Unit, Hospital Universitario Dr. Negrin, Las Palmas de Gran Canaria, Spain; 2 CIBER de Enfermedades Respiratorias, Spain; 3 Clinical and Genetic Epidemiology, Research Unit, Hospital Universitario NS de Candelaria, Tenerife, Spain

## Abstract

**Background:**

The inaccuracy of death certification can lead to the misallocation of resources in health care programs and research. We evaluated the rate of errors in the completion of death certificates among medical residents from various specialties, before and after an educational intervention which was designed to improve the accuracy in the certification of the cause of death.

**Methods:**

A 90-min seminar was delivered to seven mixed groups of medical trainees (n = 166) from several health care institutions in Spain. Physicians were asked to read and anonymously complete a same case-scenario of death certification before and after the seminar. We compared the rates of errors and the impact of the educational intervention before and after the seminar.

**Results:**

A total of 332 death certificates (166 completed before and 166 completed after the intervention) were audited. Death certificates were completed with errors by 71.1% of the physicians before the educational intervention. Following the seminar, the proportion of death certificates with errors decreased to 9% (p < 0.0001). The most common error in the completion of death certificates was the listing of the mechanism of death instead of the cause of death. Before the seminar, 56.8% listed respiratory or cardiac arrest as the immediate cause of death. None of the participants listed any mechanism of death after the educational intervention (p < 0.0001).

**Conclusion:**

Major errors in the completion of the correct cause of death on death certificates are common among medical residents. A simple educational intervention can dramatically improve the accuracy in the completion of death certificates by physicians.

## Background

Every day in Spain, 2 deaths per 100.000 inhabitants occur [1]. While data on the number of deaths in a community or a country is something precise and accurate, the identification of the causes of death is often incorrect [2,3]. Although death certification is mainly a professional activity oriented to medico-legal purposes, the information from death certificates is used for epidemiological purposes to monitor the health status of a population and direct the allocation of health care resources for health care programs and research [4-6]. The standard death certificate in Europe follows the recommendations of the World Health Organization which recommends that the underlying cause of death should be used for statistical analysis of mortality [3]. Although most physicians routinely complete death certificates as part of their professional activity and the process is well standardized, a critical review of the information contained in death certificates shows that they are full of errors [7]. Correct certification of the cause of death requires: 1) awareness of the distinction between "cause" and "mechanism" of death, and 2) understanding the meaning of the concepts "immediate cause of death" and "underlying cause of death", both of them included in the standard format of the medical section of a death certificate.

On the other hand, most published papers report a rate of 25 to 50% of discrepancies between *pre-mortem *and *post-mortem *diagnosis in hospitalised patients [8]. Considering that less than 40% of deaths occur in a hospital environment and that in less than 10% of them an autopsy is performed, current statistics could not represent the true incidence of death-causing diseases in the community. Despite its importance, there are few studies comparing the inaccuracies and errors incurred during the completion of death certificates. In this paper, we describe the impact of an educational intervention delivered to several groups of medical residents designed to improve the accuracy of death certification.

## Methods

### Educational intervention and Subjects

This study was performed over an 18-month period at various Spanish teaching hospitals. Medical trainees from various medical specialties (family medicine, internal medicine, anaesthesiology, general surgery, critical care medicine) were invited to attend a 90-min seminar on the proper completion of death certificates. There was no fee for registration. The seminars were held in a classroom in each hospital under the approval of the Continuing Medical Education Office in each institution. The seminars were delivered as interactive workshops by one the authors (JV) and were based on a previous publication with the same title written by the same author [3]. In each seminar, the following aspects were reviewed and analysed: (i) current legislation on death certification, (ii) distinction between causes and mechanisms of death, (iii) review of most common errors in the certification of the cause of death, and (iv) some recommendations to improve the quality and completion in death certificates.

### Case-scenario of death certificate

**Figure 1:**
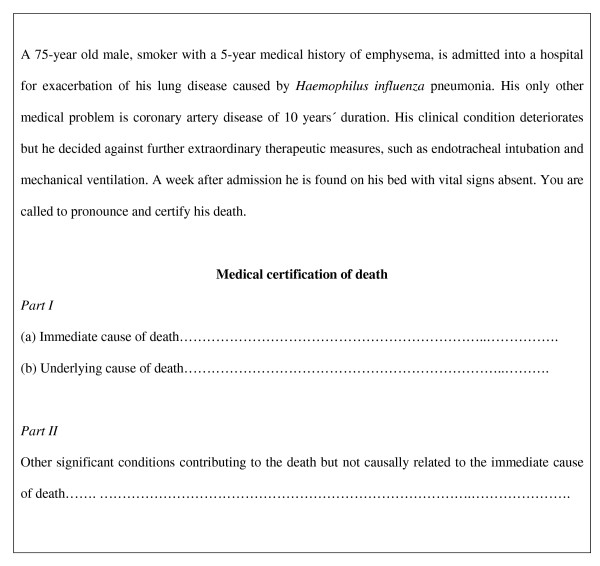
Summary of a case scenario used in a seminar to illustrate common errors in the certification of death.

Immediately before the seminar, all the attendants were invited to read carefully a case-scenario and anonymously complete a death certificate for that case (Figure 1). This case was retrieved from a similar study performed with internal medicine residents in Canada [9]. For the purpose of this study, the example of the death certificate was a simplification of the standard medical certificate of death issued by the Royal College of Physicians and Surgeons, Madrid, Spain. Once completed, the death certificates were retrieved from each participant just before the seminar took place. At the end of the seminar, and unexpected by the medical residents, the same case-scenario was asked to be completed. From all the four major possibilities of death certification (Table 1), only one of them was considered to be accurate.

**Table 1:** Four examples of possible ways of completion of death certificate for the same case scenario described in Figure 1. Only the example D is accurate.

Examples		Type of errors
**Example A**		
*Part I*		
(a)	Respiratory (or cardiac) arrest	Listing mechanisms of death without an underlying cause of death
(b)	-	
*Part II*	-	
		
**Example B**		
*Part I*		
(a)	Emphysema	Improper sequencing of events
(b)	Pneumonia	
*Part II*	Coronary artery disease	
		
**Example C**		
*Part I*		
(a)	Coronary artery disease	Listing two causally unrelated diseases
(b)	COPD	Use of abbreviations
*Part II*	-	
		
**Example D**		
*Part I*		
(a)	*H. Influenza *Pneumonia	None
(b)	Emphysema	
*Part II*	Coronary artery disease	

### Data Analysis

Death certificates were classified categorically by the presence and types of errors. Six types of errors were identified: (i) listing the mechanism of death without an underlying disease as the cause of death; (ii) improper temporal sequencing of events; (iii) listing two causally unrelated, etiologically specific diseases as the cause of death; (iv) use of abbreviations as a mean of identifying diseases, (v) listing the mechanism of death followed by the proper underlying cause of death, and (vi) listing the cause of death as one of other significant conditions contributing to the death but not causally related to the immediate cause of death. Fisher's exact test was used to compare differences in the proportion of correct or incorrect completion of death certificates before and after the educational intervention. The strength of the association between the proportions of change in the overall accuracy of the completion of death certificates (an improvement attributable to the seminar) before and after the educational intervention was expressed by the relative risks of the estimates (RR) with confidence intervals (CI). Analyses were performed by means of EPIDAT 3.0 [10]. A significance level of 5% was used.

## Results

A total of seven seminars were held in seven different cities across Spain (Table 2) with an overall attendance of 166 physicians. A total of 332 death certificates were analysed (166 before and 166 after the seminar). Before the seminar, 71.1% of the completed certificates had at least one error; less than a third of participants (28.9%) completed the death certificate correctly. Of the 118 physicians that incorrectly completed the certification of death, 67 of them (56.8%) listed respiratory arrest, cardiac arrest or cardiopulmonary arrest as the immediate or the underlying cause of death. Types and frequency of errors in the death certification before and after the educational intervention are listed in Table 3.

**Table 2:** List of hospitals and cities where a 90-min seminar on "How to improve the accuracy of death certification" was held. Number of medical residents participating in each session is listed as well. A total of 332 death certificates were audited.

**Hospital**	**City**	**No. participants**
Hospital Universitario Río Hortega	Valladolid	42
Hospital Clínico Universitario	Salamanca	40
Hospital Universitario Dr. Josep Trueta	Gerona	29
Hospital Universitario Reina Sofía	Córdoba	20
Complejo Hospitalario Xeral-Calde	Lugo	20
Hospital Marítimo de Oza	La Coruña	9
Hospital Universitário N. S. de Candelaria	Santa Cruz de Tenerife	6
	Total participants	166

**Table 3:** Types and frequency of errors in the death certification before and after the educational intervention.

**Type of Error**	**Pre-interventionn (%)**	**Post-interventionn (%)**	**p-value**
Listing the mechanism of death without an underlying disease as the cause of death	71 (42.6)	4 (2.4)	<0.0001
Improper temporal sequencing of events	31 (18.7)	1 (0.6)	<0.0001
Listing two causally unrelated, etiologically specific diseases as the cause of death	10 (6.0)	5 (3.0)	0.290
Use of abbreviations as a mean of identifying diseases	9 (5.4)	5 (3.0)	0.413
Listing the mechanism of death followed by the proper underlying cause of death	22 (13.3)	0 (0)	<0.0001
Listing the cause of death in Part II (as one of other conditions contributing to the death but not causally related to the immediate cause of death)	46 (27.7)	5 (3.0)	<0.0001

**Figure 2:**
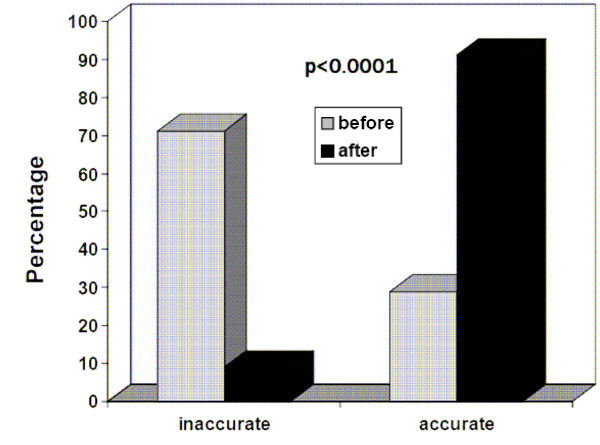
Rate of accuracy in the completion of a death certificate based on the case scenario of Figure 1, before and after a 90-min educational intervention entitled "How to improve the accuracy of certification of death". Before the seminar, 71.1% of death certificates contained at least one error and this proportion decreased to 9% after the educational intervention (p < 0.0001).

After the seminar, most physicians (91%) correctly completed both the causes of death and the temporal sequence of causes. Only 9% of the death certificates contained at least one error. None of the physicians listed respiratory arrest, cardiac arrest or cardio-respiratory arrest as a cause of death. The probability of accurately completing the death certificate after the seminar was more than three times higher than before the seminar (RR 3.67, 95%CI: 2.84–4.73) and the overall rate of improvement attributable to the educational intervention was 62.1% (p < 0.0001) (Figure 2).

## Discussion

In this study, the 71.1% proportion of death certificate with at least one error filled by medical residents is higher than the reported in previous studies. However, our study shows that a simple educational intervention can increase the accuracy rate by more than three-fold. To our knowledge, only four previous groups [9,11-13] have implemented an educational intervention to improve the accuracy of death certification and measured the changes after the intervention. In those studies, the rate of inaccuracies was lower but their study design was very different from ours. Weeramanthri et al [11] from Australia attempted to change physician behaviour on death certification by means of written guidelines. They sent written educational material as part of the questionnaire and compared rates of errors 1 month before and 1 month after the intervention. Less than 20% of physicians responded the questionnaire and although the proportion of error dropped from 22.4% to 15.1%, this change was not statistically significant. Pain et al [12] from England evaluated in a randomised controlled fashion the effectiveness of a training video on proper death certification among 185 medical students. The video was shown as an addition to the usual lecture on death certification. Although students assigned to see the video scored slightly better overall, adding this teaching method to the usual lecture had a limited effect on the overall knowledge and skills. Myers et al [9] from Canada implemented an interactive 75-min seminar on proper completion of death certificates delivered to medical residents rotating in a teaching hospital. The seminar included the discussion of 10 case scenarios accompanied by the correctly completed death certificate reviewed with the help of a coroner. In addition to the seminar, a memorandum was attached reminding residents to avoid the use of mechanisms of death in the Cause of Death section of the death certificate. The audit of 146 death certificates before the seminar for the same case-scenario showed that 32.9% of them contained at least one major error and 84.2% of them had at least one minor error. Although only 83 certificates were completed after the seminar, frequency of major errors dropped to 15.7% but the frequency of minor errors did not change significantly (90.4%). In a randomized fashion, Lakkireddy et al [13] from USA have recently evaluated in 200 internal medicine residents the impact of two educational interventions: a 45-min interactive workshop or printed instruction material. They used two case simulations that were prepared based on real-life cases, as we did, and found that before the educational intervention, only 15.5% of residents correctly identified the cause of death and 60% incorrectly identified a cardiac cause of death. Although both interventions significantly improved the appropriate completion of death certification, Lakkireddy et al demonstrated that interactive workshops are a better mode of teaching than printed handouts.

The concepts of the "underlying cause of death", the "immediate cause of death" and the "mechanism of death" are often a source of confusion for certifying physicians [14-16]. The mechanism of death is a physiologic derangement or biochemical disturbance by which a cause of death exerts its lethal effect. One of the most common errors is attributing the cause of death to respiratory arrest, cardiac arrest, cardiorrespiratory arrest, or asystole in all deaths. Except in heart donors, cardiac arrest is a meaningless term; it is simply a condition to be dead and it must not be used as an underlying cause of death. The underlying cause of death is the disease that triggered the chain of events leading to death, without which death would not have occurred. It should be as etiologically specific as possible. Non specific conditions (e.g. sepsis, haemorrhage, respiratory failure, renal failure) have more than one possible cause and are not acceptable as an underlying cause of death [17]. The immediate cause of death is the final complication resulting from the underlying cause of death which is occurring closest to the time of death and directly causing death. The certifying physician does not always have enough information to be certain of the immediate cause of death. Therefore, in many cases, an immediate cause of death may not be identifiable and an underlying cause of death can stand alone in part I of the medical section of the death certificate. "Natural causes" may be entered if the manner of death is natural and the physician does not know exactly how the patient died [18]. Using the phrase "natural causes" is preferable to making a possibly inaccurate guess as to the cause of death.

Experience does not appear to improve death certification practice. Methods to improve standards of practice and performance include early and continuing post-graduate education, and practical accessible guidance on death certification completion. Several authors [7,13-15] have found that most physicians consider accurate death certification important but they feel insufficiently instructed to correctly complete the death certificate. In a recent study, Cirera et al [19] examined the training need priorities in Spain for proper death certification and found out that the highest priorities were how to accurately declare a death and improve medical training. We think that, for example, an immediate audit of completed death certificate by skilled attending physicians is a valid method. In fact, most physicians have reported that, given that possibility, they would modify the cause of death statement in some circumstances [7]. On the other hand, pathologists could serve as a better resource for teaching clinicians given their familiarity with the death certification process.

There are some limitations to our study. First, we have used only one method to improve the accuracy of death certification, but as it has been shown by Lakkireddy et al [13], this type of interactive workshop is perhaps the most effective educational intervention. Second, the information given in the case scenario was simple and clear to exclude the possibility of incorrect clinical diagnoses that very frequently physicians face in their current medical practice. Lastly, the post-educational assessment was done immediately after the seminar and we do not know for how long the residents can keep their abilities over time. We feel that educational interventions to improve accuracy of death certificate should be mandatory not only during the residence programs but as part of the continuing medical education package for attending physicians in community health care centers and teaching hospitals. In the absence of accurate information on death causing diseases, we cannot guarantee that hospital, community or population-based mortalities can be used as a source of information to allocate health care resources and foster research where is needed to improve the health of our citizens.

## Conclusion

In summary, major errors in the completion of the correct cause of death on death certificates are very common among medical residents. We have demonstrated that a simple, 90-min seminar can dramatically improve the accuracy in the completion of death certificates by physicians. We believe that educational interventions to improve accuracy of death certificate should be mandatory not only during the residence programs but as part of the continuing medical education package for physicians.

## Competing interests

The author(s) declare that they have no competing interests.

## Authors' contributions

JV conceived the study, obtained permission for the study, delivered all the interactive workshops, collected the data, and drafted the manuscript. LPM helped in the design of the study, participated in the data storage, performed the statistical analysis, and helped to draft the manuscript. Both authors read and approved the final manuscript.

## Pre-publication history

The pre-publication history for this paper can be accessed here:


